# Supplementary material for: Diverse mechanisms of translation arrest by a Clostridia ribosome stalling peptide CliM
Source: Nat Commun. 2026 May 18;17:4202. doi: 10.1038/s41467-026-72673-5 (PMC13183943; doi:10.1038/s41467-026-72673-5)
Supplement: Supplementary file 1 — Supplementary Information [file 41467_2026_72673_MOESM1_ESM.pdf]

Supplementary Information

For

Diverse mechanisms of translation arrest by a Clostridia ribosome stalling peptide CliM

Mayu Yoshida[1,*], Felix Gersteuer[2,*], Ole Berendes[3], Keigo Fujiwara[1,4], Haaris A. Safdari[2], Helge Paternoga[2], Hiraku Takada[1,5], Nozomu Obana[6,7], Helmut Grubmüller[3], Lars Bock[3], Daniel N. Wilson[2,#], Shinobu Chiba[1,#]

[1] Faculty of Life Sciences and Institute for Protein Dynamics, Kyoto Sangyo University, Kamigamo, Motoyama, Kita-ku, Kyoto 603-8555, Japan.

[2] Institute for Biochemistry and Molecular Biology, Martin-Luther-King-Platz 6, University of Hamburg, 20146 Hamburg, Germany.

[3] Department of Theoretical and Computational Biophysics, Max Planck Institute for Multidisciplinary Sciences, Göttingen, Germany

[4] Department of Gene Function and Phenomics, National Institute of Genetics, Mishima, Japan

[5] Biotechnology Research Center and Department of Biotechnology, Toyama Prefectural University, 5180 Kurokawa, Imizu, Toyama 939-0398, Japan

[6] Transborder Medical Research Center, Institute of Medicine, University of Tsukuba, Tsukuba, Japan

[7] Microbiology Research Center for Sustainability (MiCS), University of Tsukuba, Tsukuba, Japan

* These authors contributed equally

# Corresponding authors:

Prof Shinobu Chiba (schiba@cc.kyoto-su.ac.jp)

Prof Daniel N. Wilson (Daniel.wilson@uni-hamburg.de)

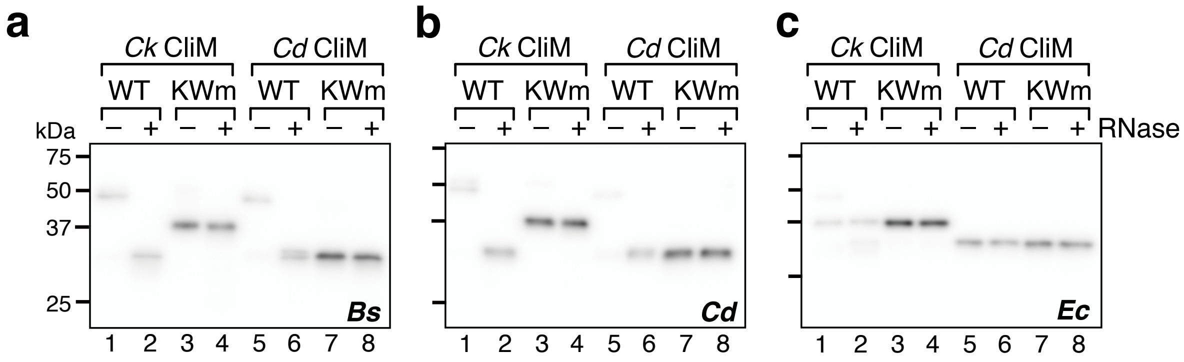

Supplementary Fig. 1: Ck and Cd CliM arrest Bs and Cd ribosomes. a-c Western blot analysis of the in vitro translation products of Ck and Cd CliM. WT or KWm mutant derivatives of the *gfp-cliM-myc-lacZα* translational fusion reporters were translated in the Bs PURE (a), Cd PURE (b), or Ec PURE (c). The products were separated in neutral-pH gels and immunoblotted using anti-GFP antibody. Samples treated with RNase A (+) were analyzed alongside untreated samples (-) to distinguish peptidyl-tRNA from full-length hydrolyzed products. Molecular size markers (kDa) are shown on the left. Western blotting was independently repeated at least twice to ensure reproducibility. Source data are provided as a Source Data file.

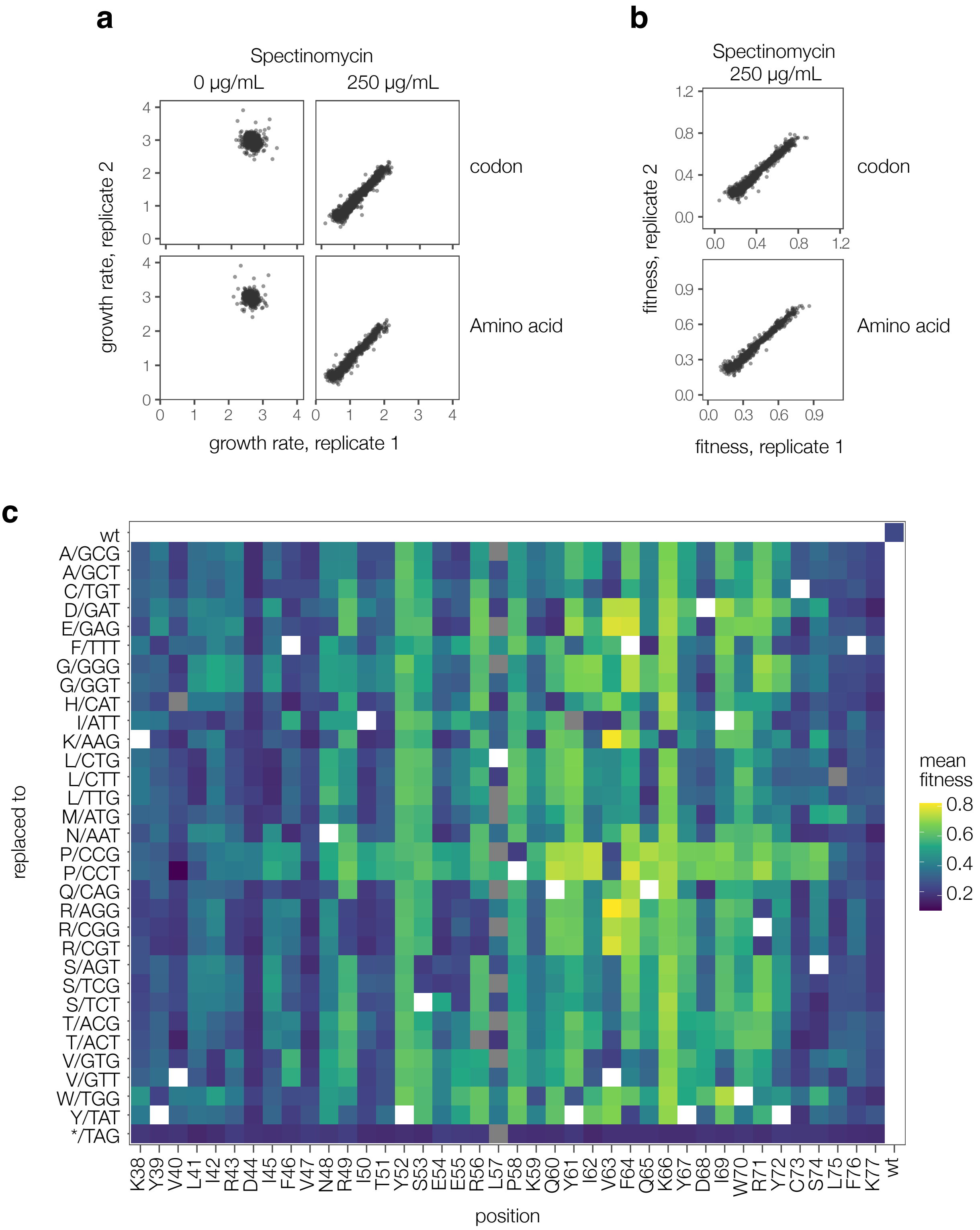

Supplementary Fig. 2: Reproducibility of Deep mutational scanning. (a-b) Scatter plots of growth rates (a) and fitness values (b) of each variant are shown at the codon level (upper) and at the amino acid level (lower), where amino acid–level values were obtained by averaging synonymous codon variants. In each plot, biological replicate 1 is shown on the x-axis and biological replicate 2 on the y-axis, demonstrating reproducibility across replicates. **(c)** The heatmap shows the relative fitness of each variant at the codon level, calculated as the mean of two biological replicates (related to Fig. 3), demonstrating that synonymous codon variants generally exhibit similar fitness values. Residue numbers and wild-type amino acids are indicated along the bottom, and substituted residues are shown along the left.

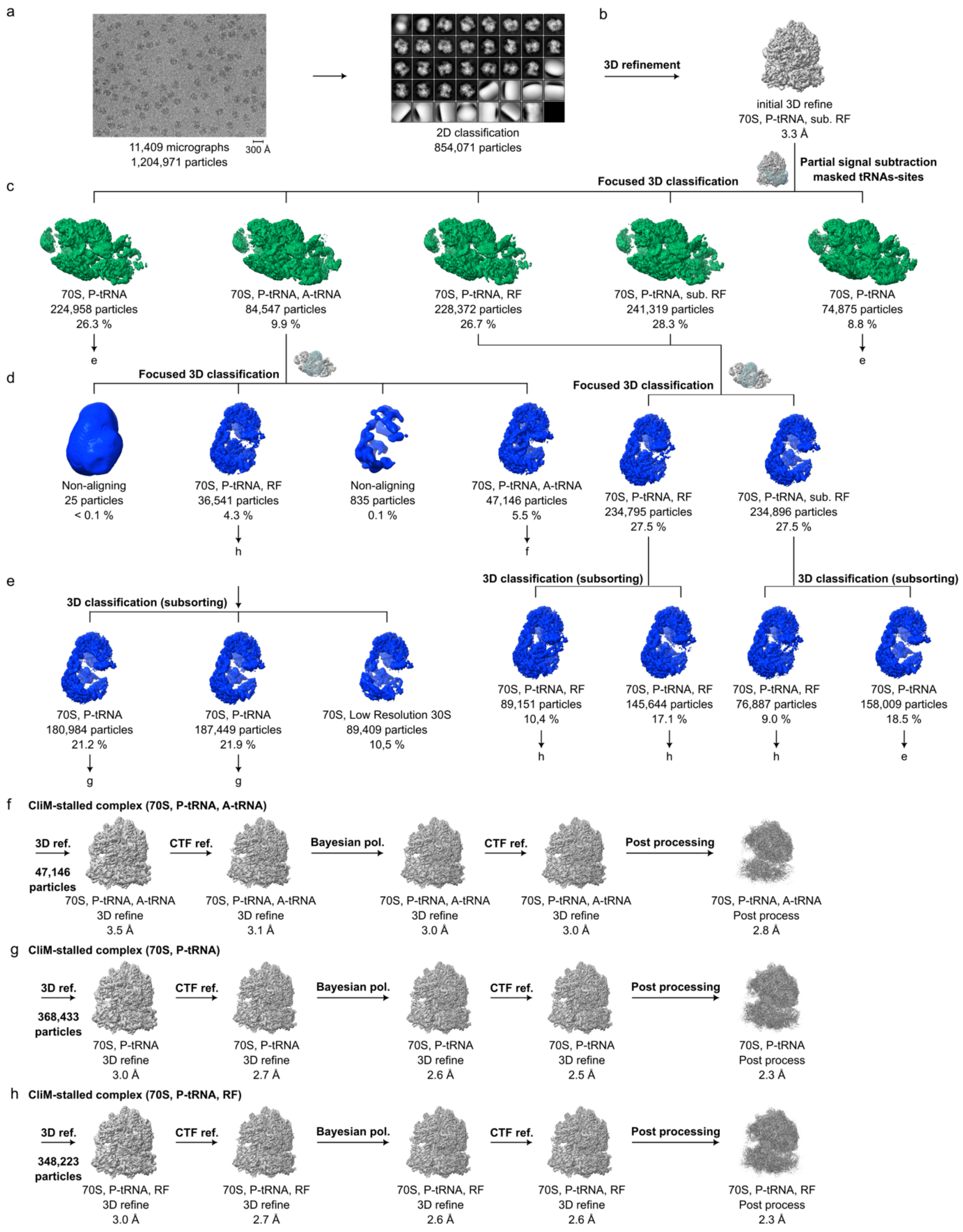

Supplementary Fig. 3: *In silico* sorting scheme for CliM. (**a**) From 11,409 micrographs, 1,204,971 particles were picked and subjected to 2D classification resulting in 854,071 ribosome-like particles. Particles were (**b**) initially 3D-refined, then (**c**) subsorted into 5 classes using a mask around the tRNA binding sites. (**d-e**) The resulting classes with P-tRNA and vacant A-site, PtRNA and release factor as well as PtRNA and A-tRNA were further subsorted with a mask around the A-site until homogeneity was reached. The minor class with (**f**) both A- and P-tRNA density (5.5%) resulted in a final resolution (at FSC 0.143) of 2.8 Å. After combining of the respective particles, two major classes with (**g**) P-tRNA and vacant A-site (43.1%) as well as (**h**) P-tRNA and release factor (40,8%) resulted in a final resolution (at FSC 0.143) of 2.3 Å, respectively.

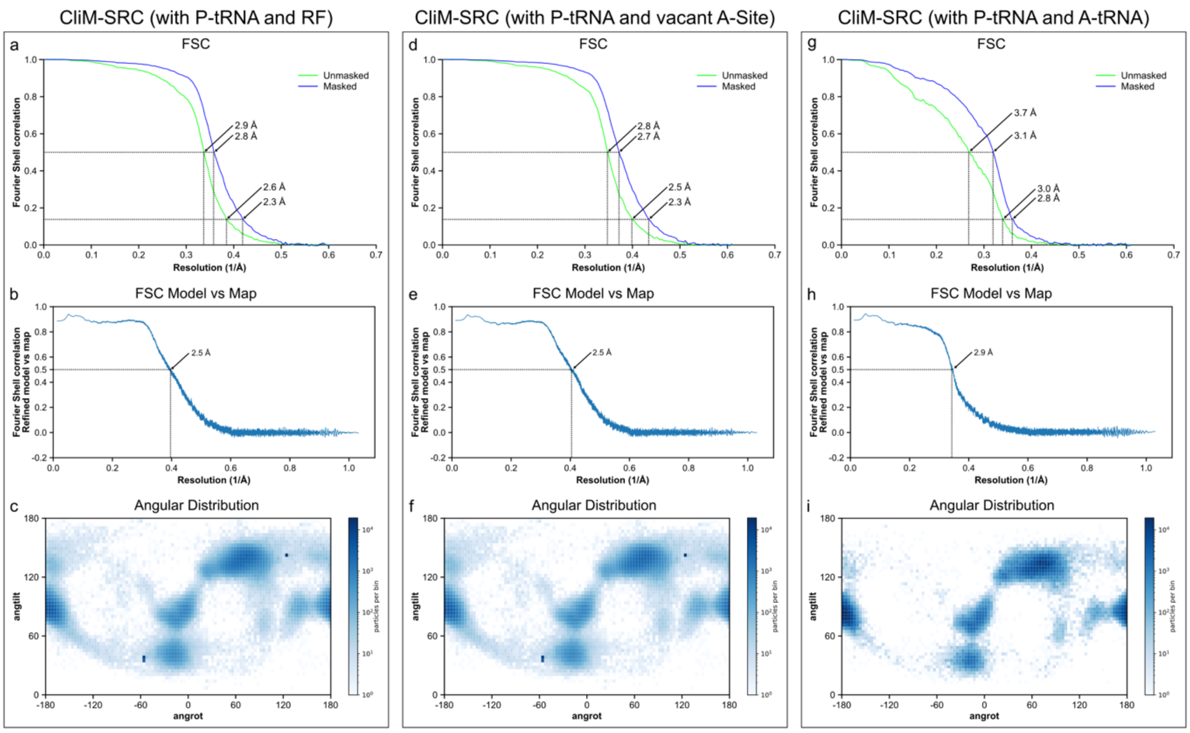

Supplementary Fig. 4: Fourier Shell Correlation curves and angular distribution for CliM-SRC. (**a-c**) Fourier shell correlation (FSC) curve of the (a) CliM-SRC containing P-tRNA and RF, (b) with P-tRNA and vacant A-site and (c) with P-tRNA and A-tRNA, with unmasked (green) and masked (blue) FSC curves plotted against the resolution (1/Å). (**d-f**) Refined model vs map FSC curve of the CliM-SRC from (a)-(c) plotted against the resolution (1/Å). (**g-i**) Angular distribution of particles used for 3D reconstruction from Relion for the CliM-SRC from (a)-(c). Particles are binned and logarithmical represented from white to blue.

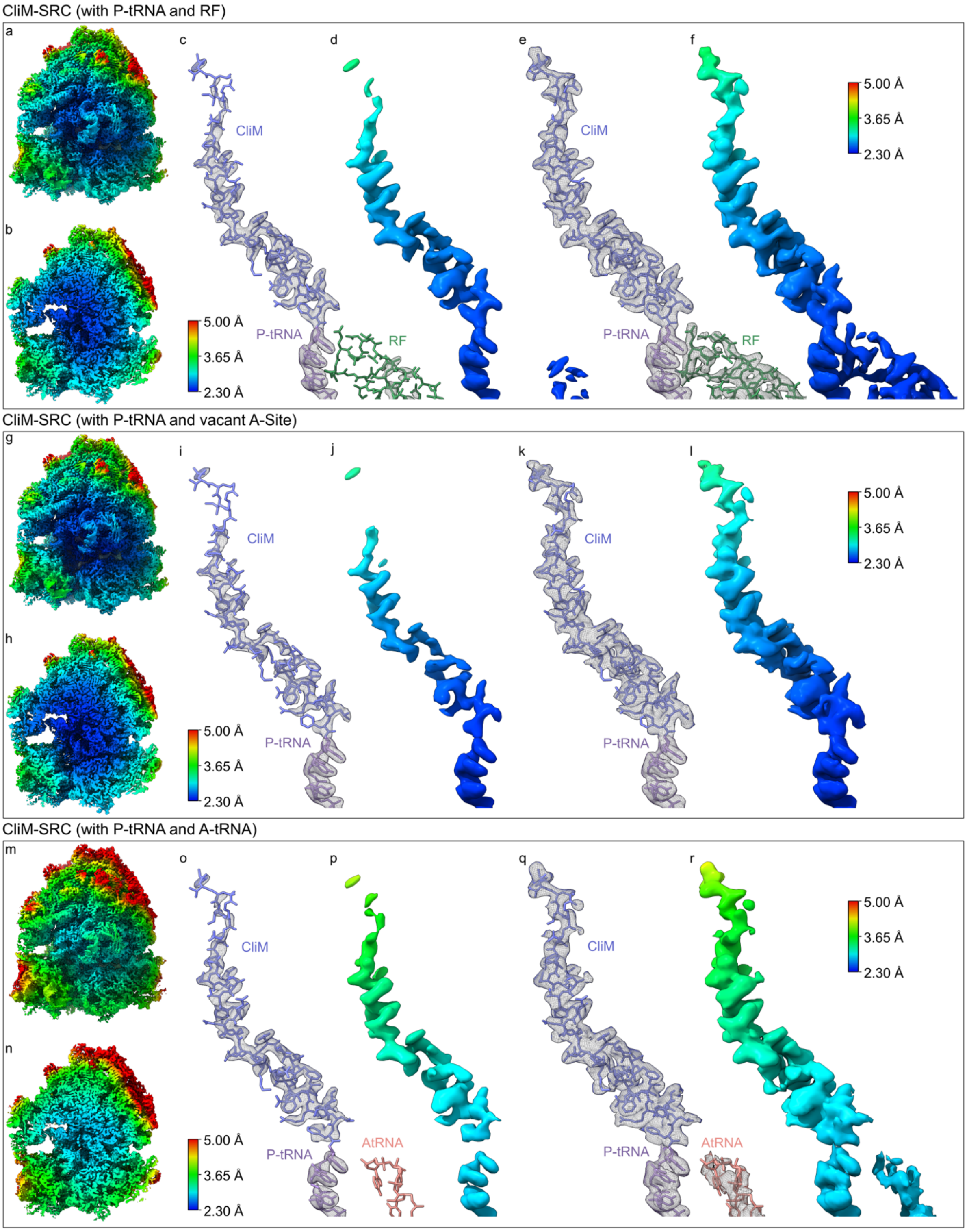

Supplementary Fig. 5: Local resolution for the CliM-SRCs. (**a-r**) Cryo-EM density for the 3D-refined map of the CliM-SRC with (**a-f**) P-tRNA and RF, (**g-l**) P-tRNA and vacant A-site, and (**m-r**) P-tRNA and A-tRNA, coloured according local resolution, or as transparent grey surface (**c, e, i, k, o, q**) with molecular model of the CliM-SRC taken from RF bound complex, and with A-tRNA (orange) in (**o,q**). In (**a,g,m**), overviews of the cryo-EM maps of the CliM-SRC are shown, whereas in (**b,h,n**), a transverse section reveals the core of the 50S subunit, including the ribosomal exit tunnel. In (**e-f, k-l, q-r**) the same representation is shown as in (**c-d, i-j, o-p**) but at a lower threshold.

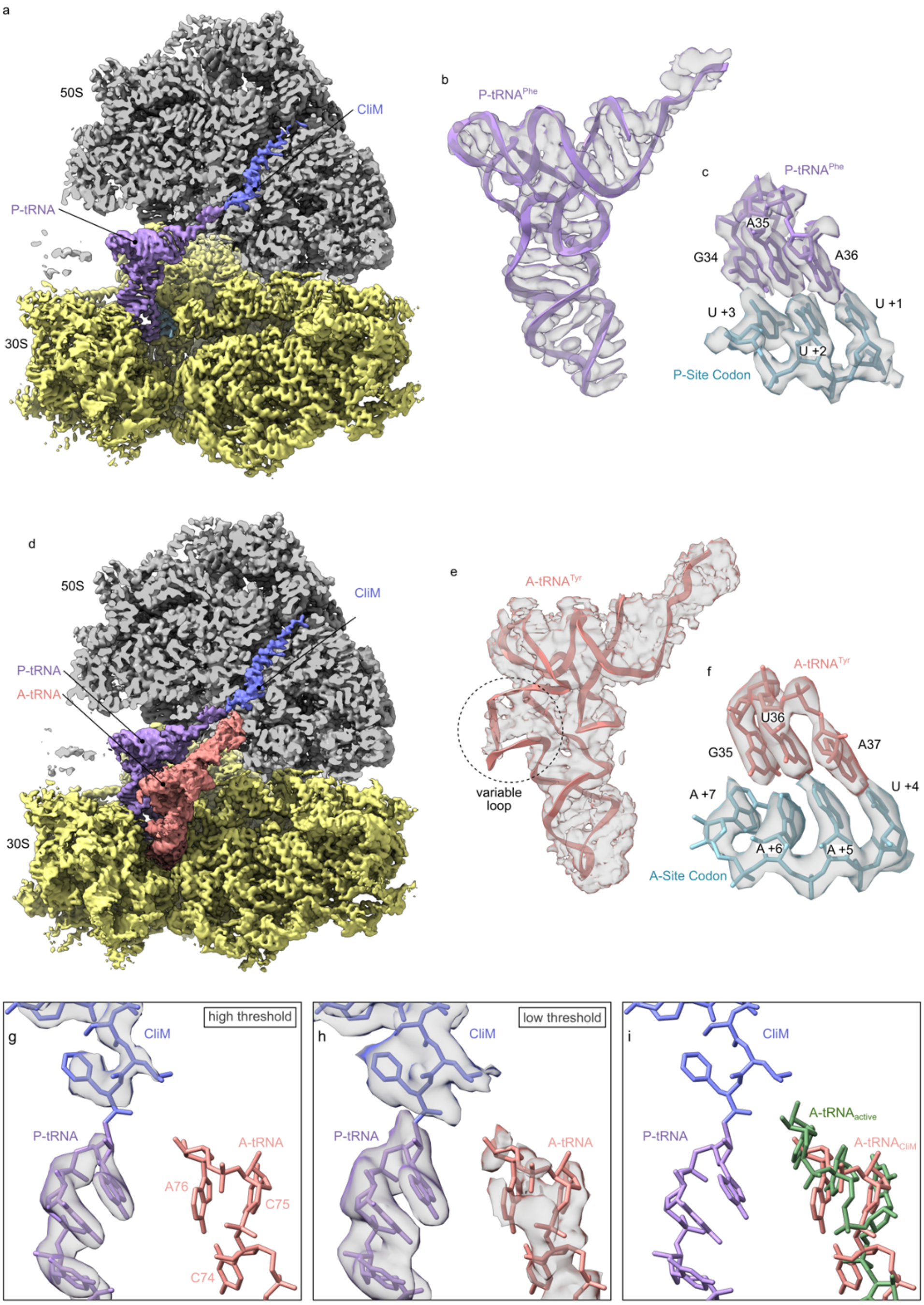

Supplementary Fig. 6: Cryo-EM density for the P-tRNA and A-tRNA. (**a**) Cryo-EM density for the 3D-refined map of the CliM-SRC (30S, yellow; 50S grey) with P-tRNA (purple) and vacant A-site, and transverse section of the 50S showing CliM nascent chain (blue) within the exit tunnel. (**b-c**) cryo-EM map density (transparent grey) for (**b**) the P-tRNA from (**a**) with fitted model for $tRNA^{Phe}$ (purple), and (**c**) anticodon of $tRNA^{Phe}$ in the P-site base-pairing with the UUU codon (positions +1 to +3) of the mRNA (green). (**d**) Cryo-EM density for the 3D-refined map of the CliM-SRC (30S, yellow; 50S grey) with P-tRNA (purple) and A-tRNA, and transverse section of the 50S showing CliM nascent chain (blue) within the exit tunnel. (**e-f**) cryo-EM map density (transparent grey) for (**e**) the A-tRNA from (**d**) with fitted model for $tRNA^{Tyr}$ (brown), and (**f**) anticodon of $tRNA^{Tyr}$ in the P-site base-pairing with the UAA stop codon (positions +4 to +6) of the mRNA (green). (**g-i**) View of the PTC of the (**g-h**) A-tRNA (rose) containing state of CliM (blue) that shows a flexible CCA-end of the A-tRNA by missing density at (**g**) high threshold, and (**h**) low threshold with noisy density, and no proper accommodation shown by the comparison with a (**i**) CCA-end of an properly accommodated tRNA (green) (PDB ID 8CVK)[1], in the A-site of the PTC.

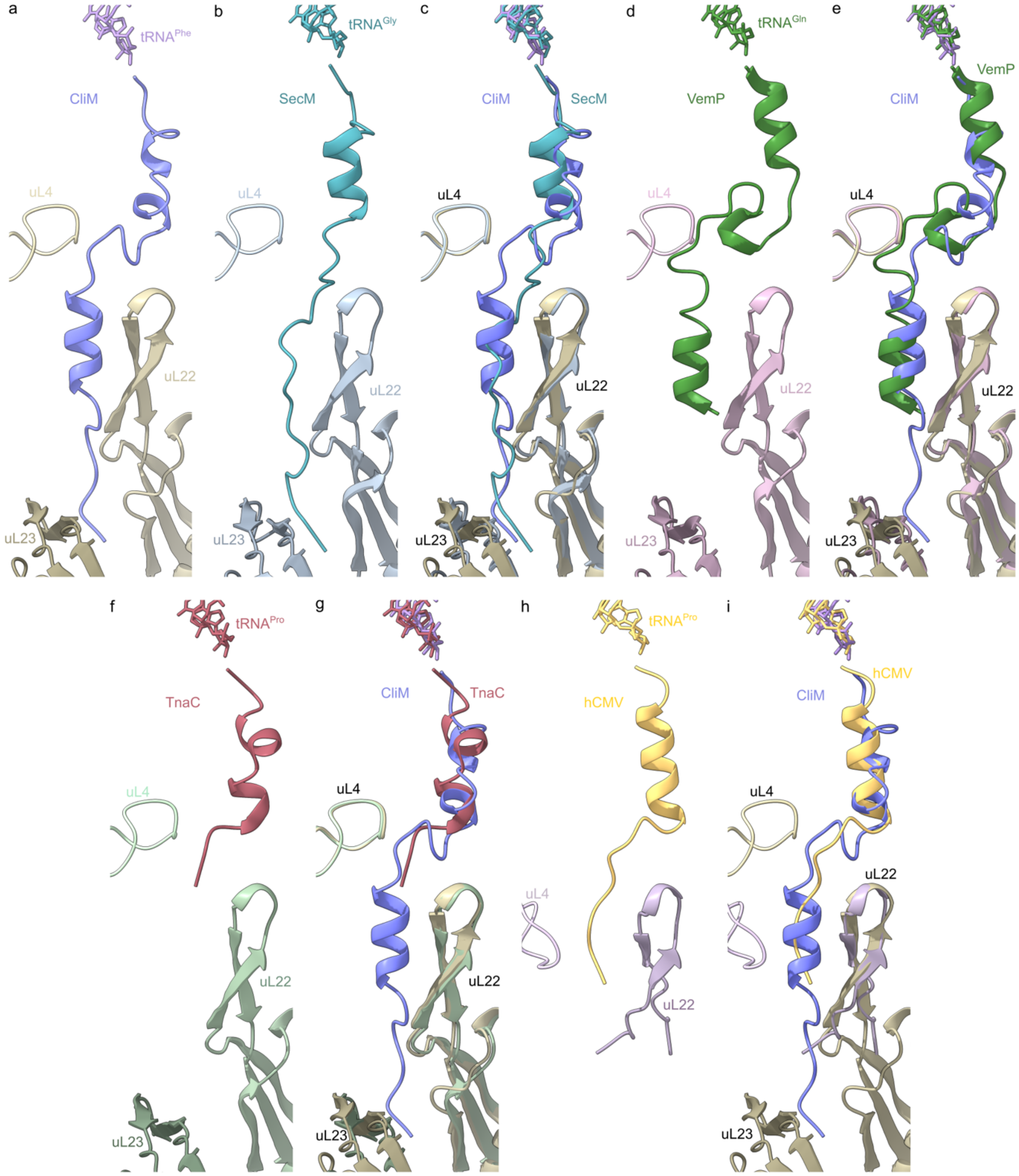

Supplementary Fig. 7: Comparison of CliM with SecM, VemP and TnaC arrest peptides. (**a**) CliM (blue) attached to the P-tRNA (lavender) in relation to uL4 (light gold), uL22 (gold) and uL23 (dark gold). (**b**) SecM (PDB ID 8QOA)[2] attached to the P-tRNA (teal) in relation to uL4 (light slate blue), uL22 (slate blue) and uL23 (dark slate blue). (**c**) Overlay (aligned on the basis of 23S rRNA) of (a) CliM and (b) SecM. (**d**) VemP (PDB ID 5NWY)[3] attached to the P-tRNA (green) in relation to uL4 (light rose), uL22 (rose) and uL23 (dark rose). (**e**) Overlay (aligned on the basis of 23S rRNA) (a) CliM and (d) VemP. (**f**) TnaC (PDB ID 7O19)[4] attached to the P-tRNA (red) in relation to uL4 (light mint), uL22 (mint) and uL23 (dark mint). (**g**) Overlay (aligned on the basis of 23S rRNA) of (a) CliM and (f) TnaC. (**h**) hCMV (PDB ID 5A8l)[5]attached to the P-tRNA (gold) in relation to uL4 (pink) and uL22 (purple). (**g**) Overlay (aligned on the basis of 23S rRNA) of (a) CliM and (h) hCMV.

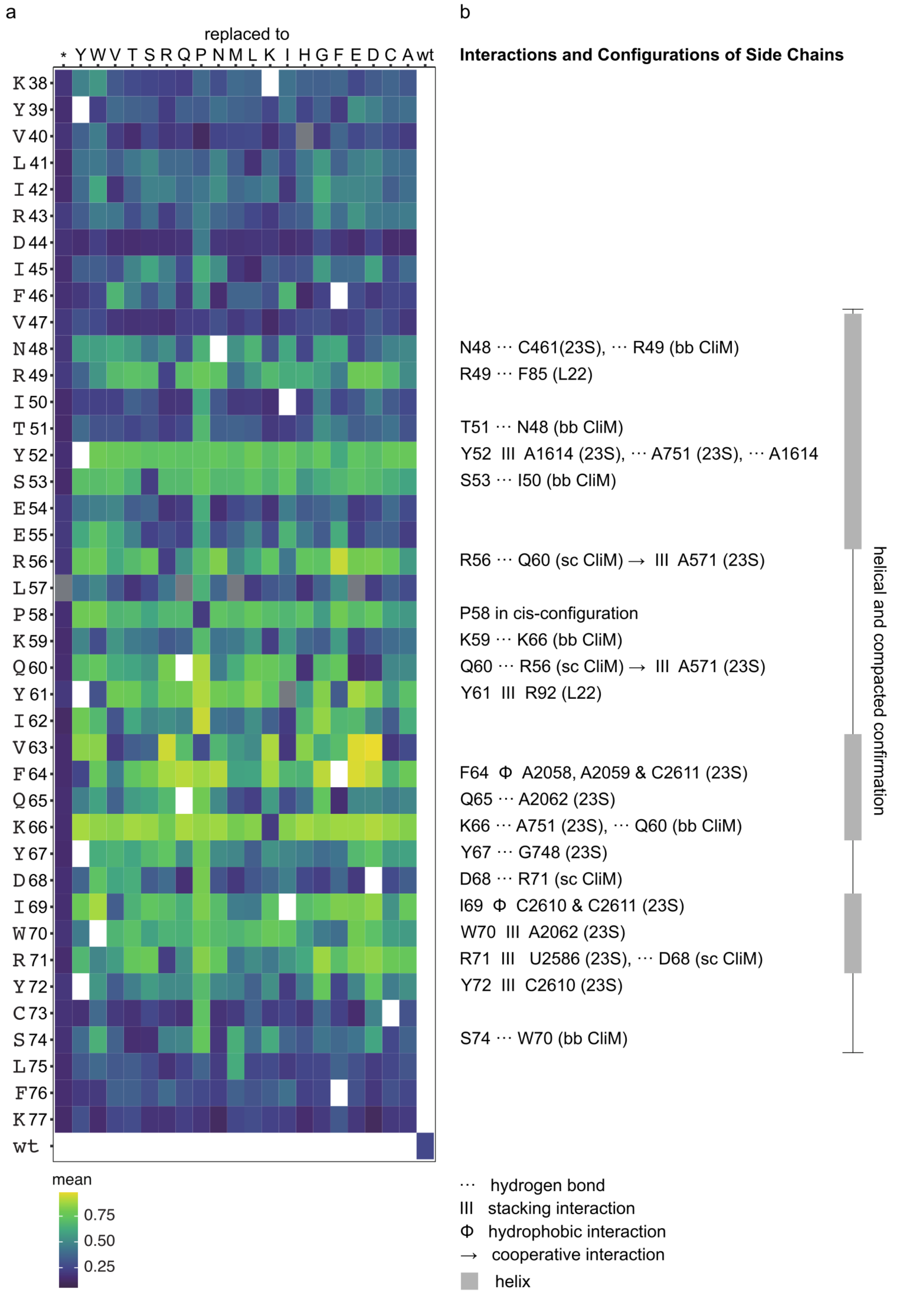

Supplementary Fig. 8: Correlation between DMS-seq and cryo-EM data for CliM. a, Heatmap from Fig 3c of relative fitness (mean of two biological replicate) of each CliM mutant compared with **b,** interactions of CliM sidechain observed in the cryo-EM structure of CliM-SRC. CliM secondary structure is also indicated.

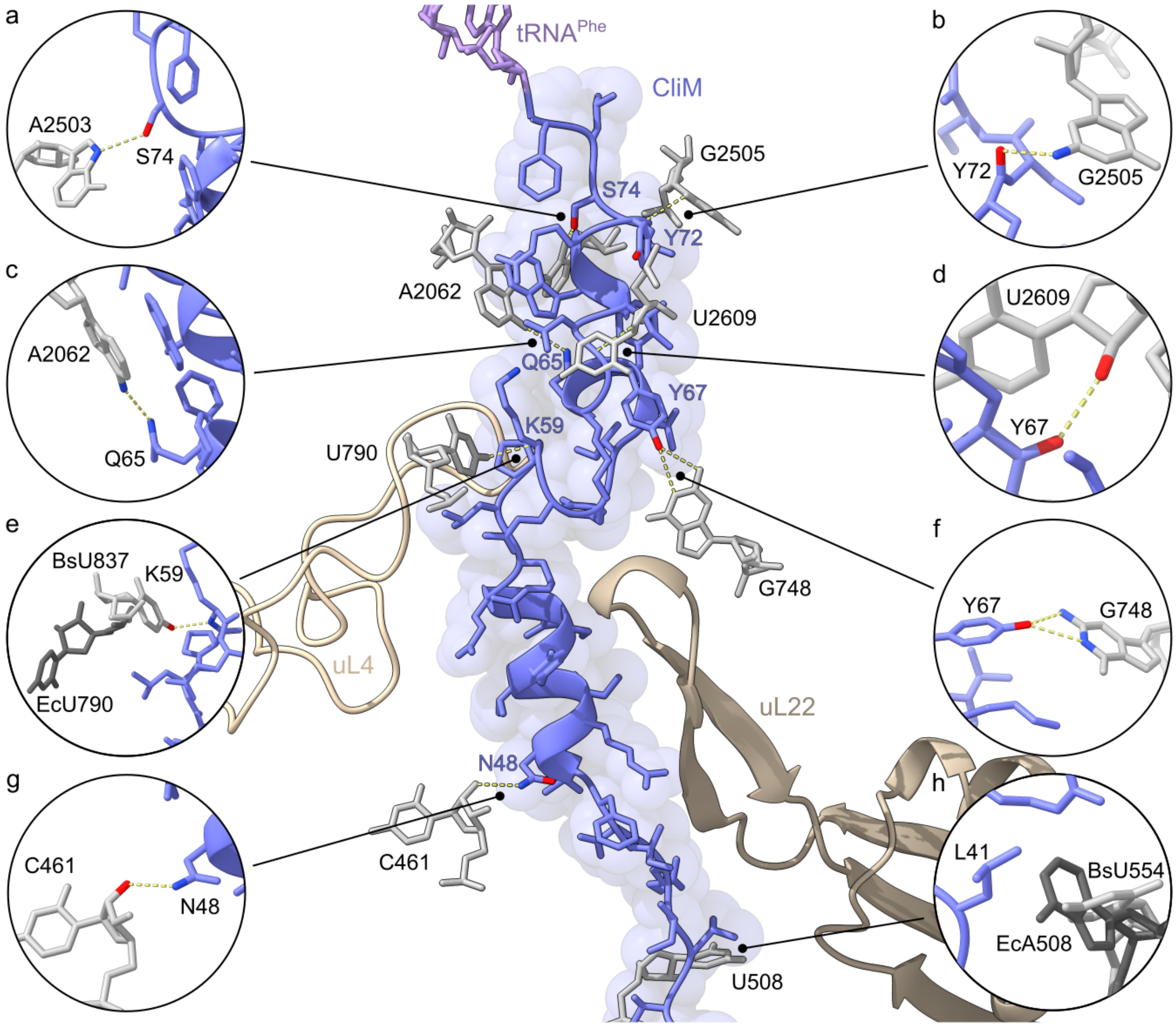

Supplementary Fig. 9: Interaction of CliM with nucleotides of the 23S rRNA. (a-h) The central panel shows the CliM nascent chain (blue) attached to the P-site tRNA (lavender) and ribosomal proteins uL4 (light gold) and uL22 (gold), with selected contacts with the 23S rRNA (grey) highlighted by individual panels. Dashed yellow lines indicated potential hydrogen bond interactions.

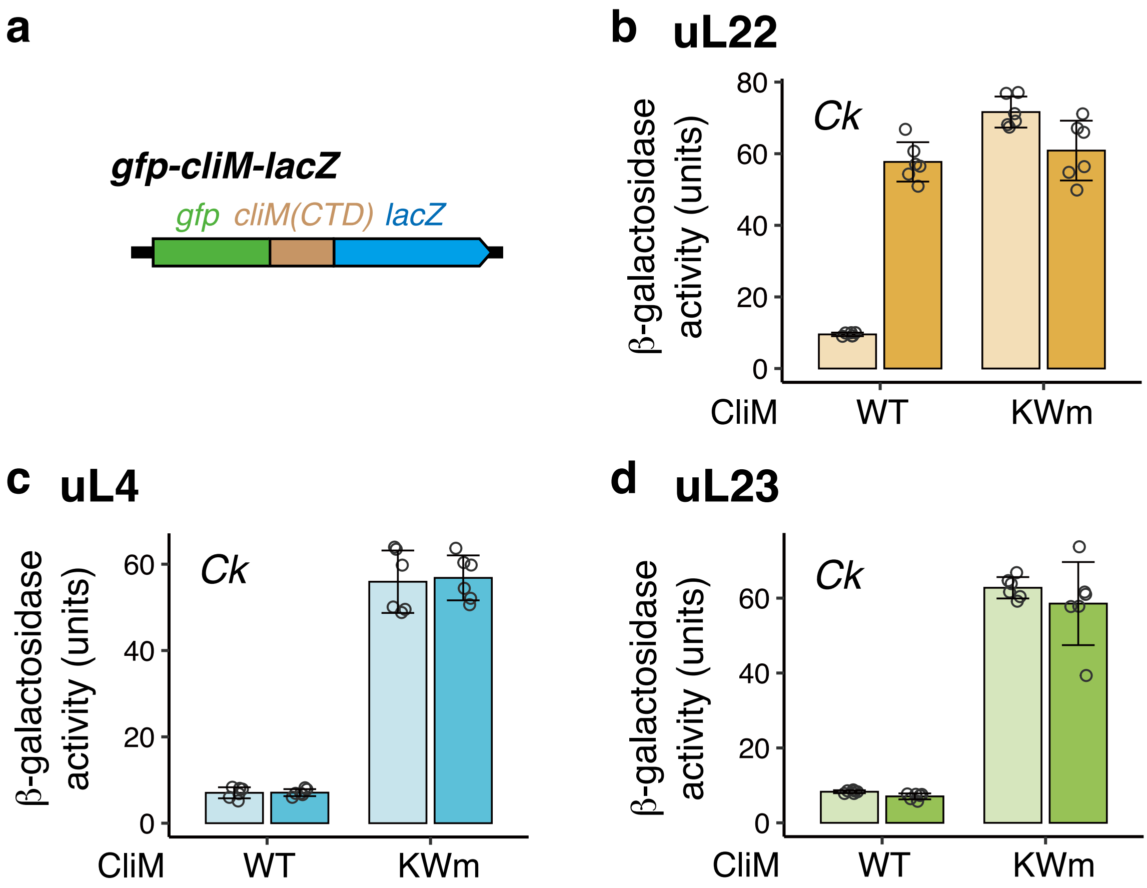

Supplementary Fig. 10: Mutation in uL22 abolishes arrest by Ck CliM. a Schematic representation of *gfp-cliM-lacZ* reporter. A gene fragment encoding the C-terminal domain (CTD) of Ck CliM was fused in-frame with *gfp* and *lacZ*. **b-d** β-galactosidase activity (mean ± s.d., n=6, biologically independent cultures) of *B. subtilis* cells carrying WT or KWm derivatives of the *gfp-'cliM-lacZ* reporter with (dirk bars) or without (light bars) loop deletions in uL22 (b), uL4 (c), or uL23 (d). Source data are provided as a Source Data file.

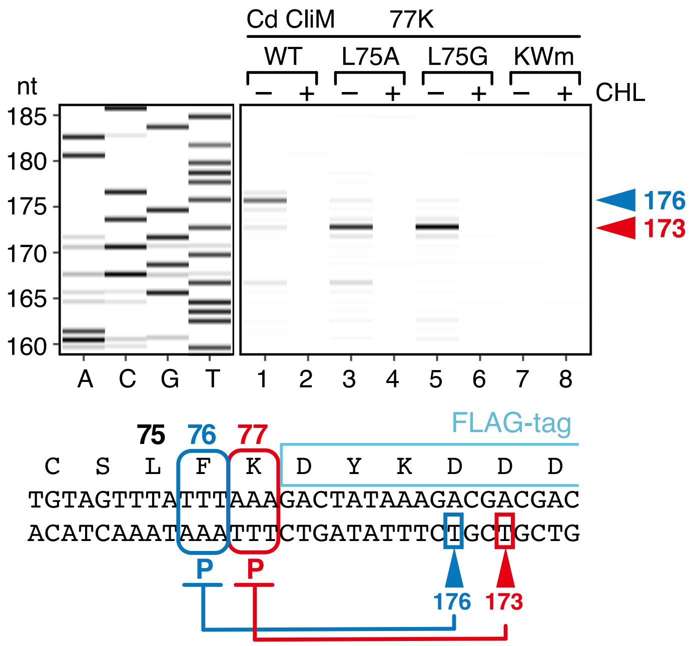

Supplementary Fig. 11: Flexible stalling site selection determined by local sequence context. Toeprinting analysis of WT and Leu75 mutant derivatives of Cd CliM with substitution of the stop codon by Lys (77K). The reverse translation products were analyzed by capillary sequencer and signals were represented as a gel-style heatmap. In vitro translation was performed in the presence or absence of chloramphenicol (CHL). The toeprint length (nt) were calibrated against dideoxy sequencing (left). The estimated stalling sites (P-site codon) based on the toeprint length are shown with their codon numbers (bottom). Toeprinting analysis was independently repeated at least twice to ensure reproducibility. Source data are provided as a Source Data file.

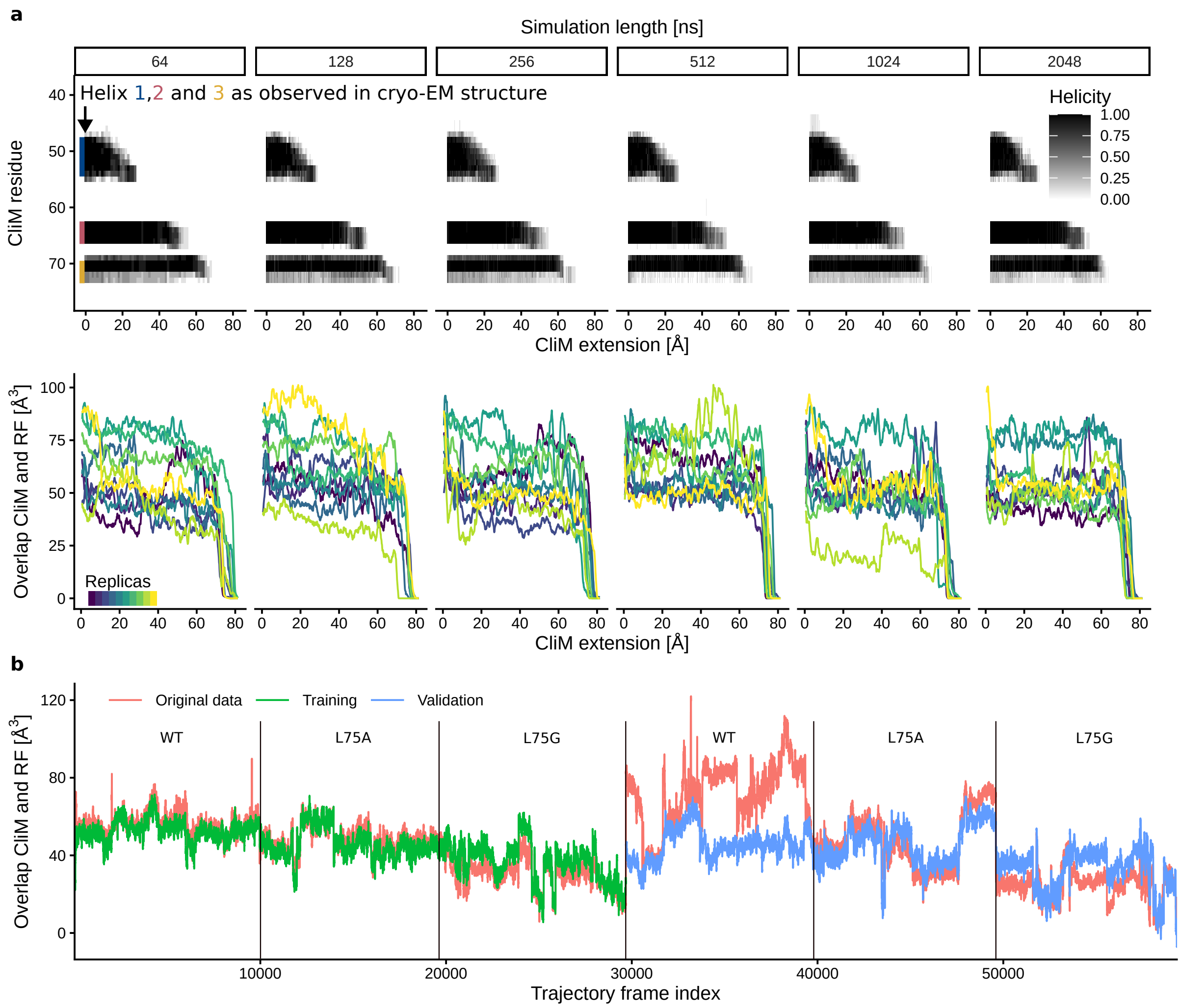

Supplementary Fig. 12: CliM dynamics during constant-velocity pulling MD simulations and cross-validation of functional mode analysis (FMA). **(a)** Top panel: For different pulling simulation lengths, the time evolution of the helicity per residue is shown. Helicity is defined as the average of secondary structure states (1: Residue part of helix, 0: Residue not part of helix) over 10 independent simulation replicas. Colored bars in the leftmost panel show the secondary structure of the cryo-EM model. Bottom panel: Time evolution of the overlap volume between CliM and aligned RF for each simulation replica. (**b**) Comparison of measured overlap volume for frames of all unbiased MD simulation trajectories (WT and L75A, L75G mutants, red line) with overlap volume predicted from FMA (green for training, blue for validation).

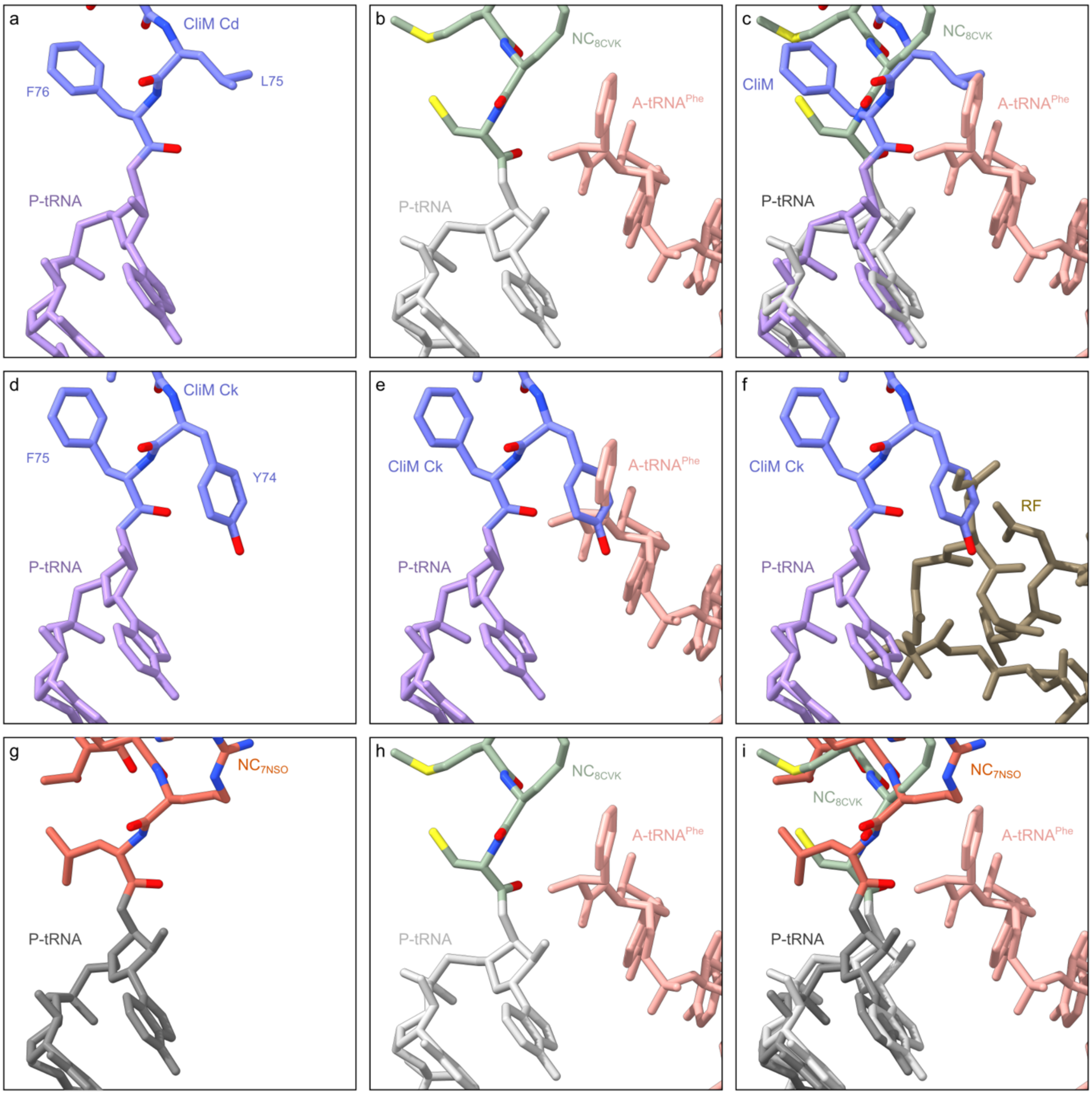

Supplementary Fig. 13: Comparison of CliM with accommodated A-tRNA. (**a**) View of the PTC of the A-tRNA containing state of CliM (blue) attached to the P-tRNA (purple). (**b**) View of the PTC of a pre-attack state (PDB ID 8CVK)[1], showing a tripeptidyl-NH-tRNA (green/light grey) at the P-site and a phenyl-NH-tRNA (rose) at the A-site. (**c**) Overlay of (**a**) and (**b**) (aligned on the basis of 23S rRNA) highlighting the incompatibility of CliM's penultimate residue Leu75 position and an accommodated aminoacyl-tRNA moiety in the A-site of the PTC. (**d**) In silico created CliM Ck (blue) mutated from CliM Cd attached to the P-tRNA (purple). (**e-f**) Overlay of (**d**) CliM Ck with (**e**) phenyl-NH-tRNA (rose) (PDB ID 8CVK)[1] and (**f**) release factor 1 (brown) (PDB ID 9MTP)[6] in the A-site, highlighting the incompatibility of proper accommodation into the A-site of the PTC for both with Tyr in the penultimate position of the CliM nascent peptide. (**g**) View of the PTC of stalled ErmDL peptide (red)(PDB ID 7NSO) [7] attached to the P-tRNA (dark grey). (**h**) Same panel as in (**b**). (**i**) Overlay of (**g**) and (**h**) (aligned of the basis of 23S rRNA) highlighting the similarity of ErmDL- and CliM-induced stalling by encroaching penultimate nascent peptide residues into the A-site that are incompatible with proper accommodation of an aminoacyl-tRNA moiety in the A-site of the PTC.

Supplementary Table 1: Strain list

strain	plasmid	host	genotype
SCB4668	pCH2713	SCB3065	spoIIIJΩkan, amyE::Ck_CliM-lacZΩcat
SCB4669	pCH2714	SCB3065	spoIIIJΩkan, amyE::Ck_CliM(dTM)-lacZ□cat
SCB4670	pCH2715	SCB3065	spoIIIJΩkan, amyE::Ck_CliM(K65A/W69A)-lacZΩcat
SCB4671	pCH2716	SCB3065	spoIIIJΩkan, amyE::Ck_CliM(dTM/K65A/W69A)-lacZΩcat
SCB4676	pCH2713	SCB2969	ΔspoIIIJ::tet, amyE::Ck_CliM-lacZΩcat
SCB4677	pCH2714	SCB2969	ΔspoIIIJ::tet, amyE::Ck_CliM(dTM)-lacZΩcat
SCB4678	pCH2715	SCB2969	ΔspoIIIJ::tet, amyE::Ck_CliM(K65A/W69A)-lacZΩcat
SCB4679	pCH2716	SCB2969	ΔspoIIIJ::tet, amyE::Ck_CliM(dTM/K65A/W69A)-lacZΩcat
SCB4696	pCH2736	SCB3065	spoIIIJΩkan, amyE::Ck_CliM-Dstem2-yidC2(1-6)-lacZΩcat
SCB4697	pCH2737	SCB3065	spoIIIJΩkan, amyE::Ck_CliM(dTM)-Dstem2-yidC2(1-6)-lacZΩcat
SCB4698	pCH2738	SCB3065	spoIIIJΩkan, amyE::Ck_CliM(K65A/W69A)-Dstem2-yidC2(1-6)-lacZΩcat
SCB4699	pCH2739	SCB3065	spoIIIJΩkan, amyE::Ck_CliM(dTM/K65A/W69A)-Dstem2-yidC2(1-6)-lacZΩcat
SCB4700	pCH2736	SCB2969	ΔspoIIIJ::tet, amyE::Ck_CliM-Dstem2-yidC2(1-6)-lacZΩcat
SCB4701	pCH2737	SCB2969	ΔspoIIIJ::tet, amyE::Ck_CliM(dTM)-Dstem2-yidC2(1-6)-lacZΩcat
SCB4702	pCH2738	SCB2969	ΔspoIIIJ::tet, amyE::Ck_CliM(K65A/W69A)-Dstem2-yidC2(1-6)-lacZΩcat
SCB4703	pCH2739	SCB2969	ΔspoIIIJ::tet, amyE::Ck_CliM(dTM/K65A/W69A)-Dstem2-yidC2(1-6)-lacZΩcat
YSB117	pKIG1225	SCB2634	rplW(d65-69)Ωkan, amyE::PmifM-gfp-Ck_cliM-lacZΩcat
YSB118	pYS37	SCB2634	rplW(d65-69)Ωkan, amyE::PmifM-gfp-Ck_cliM(K65A W69A)-lacZΩcat
YSB119	pCH2675	SCB2613	rplWΩkanWrplB, amyE::PmifM gfp-Cd_cliM(30-76)-R71CGG_L75TTA_77K-flag-lacZΩcat
YSB120	pCH2691	SCB2613	rplWΩkanWrplB, amyE::PmifM gfp-Cd_cliM(30-76)-(K66A/W70A)-R71CGG_L75TTA_77K-flag-lacZΩcat
YSB121	pCH2675	SCB2942	rplD(d66-70)Ωkan, amyE::PmifM gfp-Cd_cliM(30-76)-R71CGG_L75TTA_77K-flag-lacZΩcat
YSB122	pCH2691	SCB2942	rplD(d66-70)Ωkan, amyE::PmifM gfp-Cd_cliM(30-76)-(K66A/W70A)-R71CGG_L75TTA_77K-flag-lacZΩcat
YSB123	pCH2675	SCB2634	rplW(d65-69)Ωkan, amyE::PmifM gfp-Cd_cliM(30-76)-R71CGG_L75TTA_77K-flag-lacZΩcat
YSB124	pCH2691	SCB2634	rplW(d65-69)Ωkan, amyE::PmifM gfp-Cd_cliM(30-76)-(K66A/W70A)-R71CGG_L75TTA_77K-flag-lacZΩcat
YSB125	pCH2675	SCB2656	rpsSΩkan, amyE::PmifM gfp-Cd_cliM(30-76)-R71CGG_L75TTA_77K-flag-lacZΩcat
YSB126	pCH2691	SCB2656	rpsSΩkan, amyE::PmifM gfp-Cd_cliM(30-76)-(K66A/W70A)-R71CGG_L75TTA_77K-flag-lacZΩcat
YSB127	pCH2675	SCB2917	rpsSΩkanWrplV(d86-90), amyE::PmifM gfp-Cd_cliM(30-76)-R71CGG_L75TTA_77K-flag-lacZΩcat
YSB128	pCH2691	SCB2917	rpsSΩkanWrplV(d86-90), amyE::PmifM gfp-Cd_cliM(30-76)-(K66A/W70A)-R71CGG_L75TTA_77K-flag-lacZΩcat
YSB129	pCH2675	PY79	amyE::PmifM gfp-Cd_cliM(30-76)-R71CGG_L75TTA_77K-flag-lacZΩcat
YSB130	pCH2691	PY79	amyE::PmifM gfp-Cd_cliM(30-76)-(K66A/W70A)-R71CGG_L75TTA_77K-flag-lacZΩcat
YSB41	pKIG1225	PY79	amyE::PmifM-gfp-Ck_cliM-lacZΩcat
YSB45	pYS37	PY79	amyE::PmifM-gfp-Ck_cliM(K65A W69A)-lacZΩcat
YSB65	pYS47	SCB3065	spoIIIJΩkan, amyE::PmifM-Ck_cliM-Ck YidC2(1-6aa)-lacZΩcat
YSB66	pYS50	SCB3065	spoIIIJΩkan, amyE::PmifM-Ck_cliM(dTM)-Ck YidC2(1-6aa)-lacZΩcat
YSB67	pYS49	SCB3065	spoIIIJΩkan, amyE::PmifM-Ck_cliM(K65A W69A)-Ck YidC2(1-6aa)-lacZΩcat
YSB68	pYS51	SCB3065	spoIIIJΩkan, amyE::PmifM-Ck_cliM(dTM K65A K69A)-Ck YidC2(1-6aa)-lacZΩcat
YSB69	pYS47	SCB2969	ΔspoIIIJ::tet, amyE::PmifM-Ck_cliM-Ck YidC2(1-6aa)-lacZΩcat
YSB70	pYS50	SCB2969	ΔspoIIIJ::tet, amyE::PmifM-Ck_cliM(dTM)-Ck YidC2(1-6aa)-lacZΩcat
YSB71	pYS49	SCB2969	ΔspoIIIJ::tet, amyE::PmifM-Ck_cliM(K65A W69A)-Ck YidC2(1-6aa)-lacZΩcat
YSB72	pYS51	SCB2969	ΔspoIIIJ::tet, amyE::PmifM-Ck_cliM(dTM K65A K69A)-Ck YidC2(1-6aa)-lacZΩcat
YSB81	pKIG1225	SCB2613	rplWΩkanWrplB, amyE::PmifM-gfp-Ck_cliM-lacZΩcat
YSB82	pYS37	SCB2613	rplWΩkanWrplB, amyE::PmifM-gfp-Ck_cliM(K65A W69A)-lacZΩcat
YSB83	pKIG1225	SCB2942	rplD(d66-70)Ωkan, amyE::PmifM-gfp-Ck_cliM-lacZΩcat
YSB84	pYS37	SCB2942	rplD(d66-70)Ωkan, amyE::PmifM-gfp-Ck_cliM(K65A W69A)-lacZΩcat
YSB85	pKIG1225	SCB2656	rpsSΩkan, amyE::PmifM-gfp-Ck_cliM-lacZΩcat
YSB86	pYS37	SCB2656	rpsSΩkan, amyE::PmifM-gfp-Ck_cliM(K65A W69A)-lacZΩcat
YSB87	pKIG1225	SCB2917	rpsSΩkanWrplV(d86-90), amyE::PmifM-gfp-Ck_cliM-lacZΩcat
YSB88	pYS37	SCB2917	rpsSΩkanWrplV(d86-90), amyE::PmifM-gfp-Ck_cliM(K65A W69A)-lacZΩcat

Supplementary Table 2: Plasmid construction

plasmid	primer fw1	primer rv1	template 1	primer fw2	primer rv2	template 2	primer fw3	primer rv3	template 3	ref
pCH2126										Sakiyama 2021
pCH2527	amyE-front-fw	pDG1662-DspcR-fw	pSK69	pDG1662-DspcR-rv	FLAG_2-7-rv	pSK69	FLAG-SpcR-fw	amyE-SpcR-rv	pSK69	
pCH2612	flag-fw	gfp238-rv	pCH2527	gfp238-fw	Cdif_KYxIW-K-flag-rv	pKIG1283				
pCH2674	CliM-R71CGG-L75TTA-FLAG-fw	CliM-R71CGG-rv	pCH2612							
pCH2675	flag-fw	gfp238-rv	pCH2126	gfp238-fw	flag_2-7-rv	pCH2674				
pCH2691	Cd_CliM-repro-KWm-fw	Cd_CliM-KWm-rv	pCH2675							
pCH2713	CliM-LacZ nonTGA fw	Ck non TGA LacZ rv	pYS48							
pCH2714	CliM-LacZ nonTGA fw	Ck non TGA LacZ rv	pYS49							
pCH2715	CliM-LacZ nonTGA fw	Ck non TGA LacZ rv	pYS50							
pCH2716	CliM-LacZ nonTGA fw	Ck non TGA LacZ rv	pYS51							
pCH2730	Cd_CliM-RE-77STP-FL-fw	Cd_CliM_RE-S74-rv	pCH2675							
pCH2731	Cd_CliM-RE-L75A-77STP-FL-fw	Cd_CliM_RE-S74-rv	pCH2675							
pCH2732	Cd_CliM-RE-L75G-77STP-FL-fw	Cd_CliM_RE-S74-rv	pCH2675							
pCH2736	Ck_CliM-Dstem2-fw	Ck_CliM-Dstem2-rv	pCH2717							
pCH2737	Ck_CliM-Dstem2-fw	Ck_CliM-Dstem2-rv	pCH2718							
pCH2738	Ck_CliM-Dstem2-fw	Ck_CliM-Dstem2-rv	pCH2719							
pCH2739	Ck_CliM-Dstem2-fw	Ck_CliM-Dstem2-rv	pCH2720							
pCH2764	Cd_CliM-RE-76stp-FL-fw	Cd_CliM-C73-rv	pCH2730							
pCH2765	Cd_CliM-S74L-76stp-FL-fw	Cd_CliM-C73-rv	pCH2730							
pCH2767	Cd_CliM-S74G-76stp-FL-fw	Cd_CliM-C73-rv	pCH2730							
pCH2768	Cd_CliM-S74V-76stp-FL-fw	Cd_CliM-C73-rv	pCH2730							
pCH2774	Cd_CliM-RE-L75DKK-77STP-FL-fw	Cd_CliM_RE-S74-rv	pCH2730							
pCH2779	Cd_CliM-RE-L75S-77STP-FL-fw	Cd_CliM_RE-S74-rv	pCH2730							
pCH2780	Cd_CliM-RE-L75I-77STP-FL-fw	Cd_CliM_RE-S74-rv	pCH2730							
pCH2781	Cd_CliM-RE-L75V-77STP-FL-fw	Cd_CliM_RE-S74-rv	pCH2730							
pKIG1225										Fujiwara 2024
pKIG1283	Cd_AP_wt fw	Cd_AP_wt rv	chrDNA of C. difficile 630	myc-lacZ-fw	gfp238-rv	pSK69				
pKIG1284	Cd_AP_wt fw	Cd_AP_stp77A rv	chrDNA of C. difficile 630	myc-lacZ-fw	gfp238-rv	pSK69				
pKIG1285	Cd_AP_wt fw	Cd_AP_stp77K rv	chrDNA of C. difficile 630	myc-lacZ-fw	gfp238-rv	pSK69				
pKIG1288	amp121-128(TM62)	Cdif AP Q65 rv	pKIG1283	Cdif AP K66A W70A fw	amp121-128(TM62) antisense	pKIG1283				
pSK69										Sakiyama 2021
pYS37	amp121-128(TM62)	uYidC-Clo_klu-E64 rv	pKIG1225	uYidC_Clo_klu_K65A-W69A	amp121-128(TM62) antisense	pKIG1225				
pYS38	amp121-128(TM62)	Cd_AP_F76stp rv	pKIG1283	myc24 fw	amp121-128(TM62) antisense	pKIG1283				
pYS39	amp121-128(TM62)	Cd_AP_F75stp rv	pKIG1283	myc24 fw	amp121-128(TM62) antisense	pKIG1283				
pYS40	amp121-128(TM62)	Ck_AP_F76stp rv	pKIG1225	myc24 fw	amp121-128(TM62) antisense	pKIG1225				
pYS41	amp121-128(TM62)	Ck_AP_F75stp rv	pKIG1225	myc24 fw	amp121-128(TM62) antisense	pKIG1225				
pYS42	amp121-128(TM62)	Ck_AP_F74stp rv	pKIG1225	myc24 fw	amp121-128(TM62) antisense	pKIG1225				
pYS47	C-kluyv-pCH746-fw	C-kluyv-pCH746 rv	uYidC-Clost_kluyv (GeneArt Strings DNA Fragments)	lacZ Ile4-fw	PmifM-16nt 27rv	pCH746				
pYS49	amp121-128(TM62)	uYidC-Clo_klu-E64 rv	pYS47	uYidC_Clo_klu_K65A-W69A	amp121-128(TM62) antisense	pYS47				
pYS50	amp121-128(TM62)	C_kluyv dele_TM12-21 rv	pYS47	C_kluyv dele_TM12-21 fw	amp121-128(TM62) antisense	pYS47				
pYS51	amp121-128(TM62)	uYidC-Clo_klu-E64 rv	pYS49	uYidC_Clo_klu_K65A-W69A	amp121-128(TM62) antisense	pYS49				

Supplementary Table 3: Primer list

Primer name	Sequence (5'-3')
amp121-128(TM62)	GCAGTGCTGCCATAACCATGAGTG
amp121-128(TM62) antisense	CACTCATGGTTATGGCAGCACTGC
myc24 fw	GAACAAAAACTCATCTCAGAAGAG
uYidC_Clo_klu_K65A-W69A	CCTAAAGACTATTTGGTCTATGAAGCATATAGAATAGCATGGTATTTTGTATATTTTAAA
uYidC-Clo_klu-E64 rv	TTCATAGACCAAATAGTCTTTAGGATA
Cd_AP_F76stp rv	ATCCTCTTCTGAGATGAGTTTTTGTTCTTACAAACTACAATACCTCCAAATATC
Cd_AP_F75stp rv	ATCCTCTTCTGAGATGAGTTTTTGTTCTTAACTACAATACCTCCAAATATCATA
Ck_AP_F76stp rv	ATCCTCTTCTGAGATGAGTTTTTGTTCTTAAAAATATACAAAATACCACCATAT
Ck_AP_F75stp rv	ATCCTCTTCTGAGATGAGTTTTTGTTCTTAATATACAAAATACCACCATATTCT
Ck_AP_F74stp rv	ATCCTCTTCTGAGATGAGTTTTTGTTCTTATACAAAATACCACCATATTCTATA
lacZ Ile4-fw	ATTACGGATTCACTGGCCGT
PmifM-16nt 27rv	GCTTCATTTTACTATATGTACAAGCTG
C-kluyv-pCH746-fw	TACATATAGTAAAATGAAGCTATTGAATGGATGTGATGAAAATAGATACTCTATTAATAA
C-kluyv-pCH746 rv	ACGGCCAGTGAATCCGTAATCATGGTATTTAAAATAATGTTCATATAAATCCTCTCCTTA
C_kluyv dele_TM12-21 fw	ATGAAAATAGATACTCTATTAATAATGCAAATTCTGCTTTATGTTAGCACCATC
C_kluyv dele_TM12-21 rv	AATTTGCATTATTAATAGAGTATCTATTTTCAT
PT7-RBSkf-GFP	TAACTTTAAGAAGGAGGGAGATATACCAATGACAATGTTTGTGGGATC
lacZ60-TAATAA-21-rv	TGGTGCCGGAAACCAGGCAAATTATTAGCGCCATTCGCCATTCAGGCT
Universal primer-77-PURE	GAAATTAATACGACTCACTATAGGGAGACCACAACGGTTTCCCTCTAGAAATAATTTTGTTTAACTTTAAGAAGGAG
amyE-front-fw	TAGAGATCCGATCAGACCAGT
flag-fw	GACTATAAAGACGACGACGAC
CliM-R71CGG-L75TTA-FLAG-fw	ATTTGGCGGTATTGTAGTTTATTTAAAGACTATAAAGAC
Cd_CliM-repro-KWm-fw	TTTCAGGCATATGATATTGCGCGGTATTGTAGTTTATTT
CliM-LacZ nonTGA fw	ATTTTAAAATAAGAACCATGATTACGGATTCACTG
Cd_CliM-RE-77STP-FL-fw	GATATTTGGCGGTATTGTAGTTTATTTTAAGACTATAAAGACGAC
Cd_CliM-RE-L75A-77STP-FL-fw	GATATTTGGCGGTATTGTAGTGCATTTTAAGACTATAAAGACGAC
Cd_CliM-RE-L75G-77STP-FL-fw	GATATTTGGCGGTATTGTAGTGGATTTTAAGACTATAAAGACGAC
Ck_CliM-Dstem2-fw	GTATTTTGTATATTTTAAAATTCGTTGAACAAGTAATATTTAGGAA
Cd_CliM-RE-76stp-FL-fw	GATATTTGGCGGTATTGTAGTTTATAATAAGACTATAAAGACGAC
Cd_CliM-S74L-76stp-FL-fw	GATATTTGGCGGTATTGTCTTTTATAATAAGACTATAAAGACGAC
Cd_CliM-S74G-76stp-FL-fw	GATATTTGGCGGTATTGTGGTTTATAATAAGACTATAAAGACGAC
Cd_CliM-S74V-76stp-FL-fw	GATATTTGGCGGTATTGTGTTTTATAATAAGACTATAAAGACGAC
Cd_CliM-RE-L75DKK-77STP-FL-fw	GATATTTGGCGGTATTGTAGTDKKTTTTAAGACTATAAAGACGAC
Cd_CliM-RE-L75S-77STP-FL-fw	GATATTTGGCGGTATTGTAGTTCATTTTAAGACTATAAAGACGAC
Cd_CliM-RE-L75I-77STP-FL-fw	GATATTTGGCGGTATTGTAGTATATTTTAAGACTATAAAGACGAC
Cd_CliM-RE-L75V-77STP-FL-fw	GATATTTGGCGGTATTGTAGTGTATTTTAAGACTATAAAGACGAC
pDG1662-DspcR-fw	AATCAACGAGGTGAAATCGCTAATTTTATTGCAATAACA
gfp238-rv	TTTGTATAGTTCATCCATGCC
CliM-R71CGG-rv	ACTACAATACCGCCAAATATCATATTTCTGAAAAACTATATACTG
Cd_CliM-KWm-rv	CGCAATATCATATGCCTGAAAAACTATATACTGTTTAGG
Ck non TGA LacZ rv	CCGTAATCATGGTTCTTATTTTAAAATATACAAAATACCA
Cd_CliM_RE-S74-rv	ACTACAATACCGCCAAATATCATATTT
Ck_CliM-Dstem2-rv	ATTTTAAAATATACAAAATACCA
Cd_CliM-C73-rv	ACAATACCGCCAAATATCATATTTCTG
pDG1662-DspcR-rv	TATTGCAATAAAATTAGCGATTTCACCTCGTTGATTATG
gfp238-fw	GGCATGGATGAACTATACAAA
FLAG_2-7-rv	TTTGTCGTCGTCGTCTTTATA
Cdif_KYxIW-K-flag-rv	GTCGTCGTCTTTATAGTCTTTAAACAAACTACAATACCTCCAAAT
FLAG-SpcR-fw	TATAAAGACGACGACGACAAAAGCAATTTAATTAACGGAAAA
amyE-SpcR-rv	ACTGGTCTGATCGGATCTCTACTAATTGAGAGAAGTTTCTAT
Cd_AP_wt fw	GGCATGGATGAACTATACAAAAAAGACCTCTTAAATCATAAAATTAAGTATGTT
Cd_AP_wt rv	ATCCTCTTCTGAGATGAGTTTTTGTTCTTAAAACAAACTACAATACCTCCAAATATC
Cd_AP_stp77A rv	ATCCTCTTCTGAGATGAGTTTTTGTTCTGCAAACAAACTACAATACCTCCAAAT
Cd_AP_stp77K rv	ATCCTCTTCTGAGATGAGTTTTTGTTCCTTAAACAAACTACAATACCTCCAAATATC
Cdif AP Q65 rv	CTGAAAAACTATATACTGTTTAGGCAG
Cdif AP K66A W70A fw	CTGCCTAAACAGTATATAGTTTTTCAGGCATATGATATTGCGAGGTATTGTAGTTTGTTTTAAGAA

Supplementary Table 4: Degenerate primers for DMS

Primer name	Sequence (5'-3')
Cdif_KYxIw_K77-NNK-fw2nd	TGGCGGTATTGTAGTTTATTTNNKGACTATAAAGACGAC
Cdif_KYxIw_F76-NNK-fw2nd	ATTTGGCGGTATTGTAGTTTANNKAAAGACTATAAAGAC
Cdif_KYxIw_L75-NNK-fw2nd	GATATTTGGCGGTATTGTAGTNNKTTTAAAGACTATAAA
Cdif_KYxIw_S74-NNK-fw2nd	TATGATATTTGGCGGTATTGTNNKTTATTTAAAGACTAT
Cdif_KYxIw_C73-NNK-fw2nd	AAATATGATATTTGGCGGTATNNKAGTTTATTTAAAGAC
Cdif_KYxIw_Y72-NNK-fw2nd	CAGAAATATGATATTTGGCGGNNKTGTAGTTTATTTAAA
Cdif_KYxIw_R71-NNK-fw2nd	TTTCAGAAATATGATATTTGGNNKTATTGTAGTTTATTT
Cdif_KYxIw_W70-NNK-fw2nd	GTTTTTCAGAAATATGATATTNNKCGGTATTGTAGTTTA
Cdif_KYxIw_I69-NNK-fw2nd	ATAGTTTTTCAGAAATATGATNNKTGGCGGTATTGTAGT
Cdif_KYxIw_D68-NNK-fw2nd	TATATAGTTTTTCAGAAATATNNKATTTGGCGGTATTGT
Cdif_KYxIw_Y67-NNK-fw2nd	CAGTATATAGTTTTTCAGAAANNKGATATTTGGCGGTAT
Cdif_KYxIw_K66-NNK-fw2nd	AAACAGTATATAGTTTTTCAGNNKTATGATATTTGGCGG
Cdif_KYxIw_Q65-NNK-fw	CCTAAACAGTATATAGTTTTTNNKAAATATGATATTTGG
Cdif_KYxIw_F64-NNK-fw	CTGCCTAAACAGTATATAGTTNNKCAGAAATATGATATT
Cdif_KYxIw_V63-NNK-fw	CGACTGCCTAAACAGTATATANNKTTTCAGAAATATGAT
Cdif_KYxIw_I62-NNK-fw	GAACGACTGCCTAAACAGTATNNKGTTTTTCAGAAATAT
Cdif_KYxIw_Y61-NNK-fw	GAAGAACGACTGCCTAAACAGNNKATAGTTTTTCAGAAA
Cdif_KYxIw_Q60-NNK-fw	TCTGAAGAACGACTGCCTAAANNKTATATAGTTTTTCAG
Cdif_KYxIw_K59-NNK-fw	TATTCTGAAGAACGACTGCCTNNKCAGTATATAGTTTTT
Cdif_KYxIw_P58-NNK-fw	ACATATTCTGAAGAACGACTGNNKAAACAGTATATAGTT
Cdif_KYxIw_L57-NNK-fw	ATTACATATTCTGAAGAACGANNKCCTAAACAGTATATA
Cdif_KYxIw_R56-NNK-fw	AGAATTACATATTCTGAAGAANNKCTGCCTAAACAGTAT
Cdif_KYxIw_E55-NNK-fw	AATAGAATTACATATTCTGAANNKCGACTGCCTAAACAG
Cdif_KYxIw_E54-NNK-fw	GTAAATAGAATTACATATTCTNNKGAACGACTGCCTAAA
Cdif_KYxIw_S53-NNK-fw	TTTGTAAATAGAATTACATATNNKGAAGAACGACTGCCT
Cdif_KYxIw_Y52-NNK-fw	ATATTTGTAAATAGAATTACANNKTCTGAAGAACGACTG
Cdif_KYxIw_T51-NNK-fw	GACATATTTGTAAATAGAATTNNKTATTCTGAAGAACGA
Cdif_KYxIw_I50-NNK-fw	AGAGACATATTTGTAAATAGANNKACATATTCTGAAGAA
Cdif_KYxIw_R49-NNK-fw	ATAAGAGACATATTTGTAAATNNKATTACATATTCTGAA
Cdif_KYxIw_N48-NNK-fw	TTAATAAGAGACATATTTGTANNKAGAATTACATATTCT
Cdif_KYxIw_V47-NNK-fw	GTTTTAATAAGAGACATATTTNNKAATAGAATTACATAT
Cdif_KYxIw_F46-NNK-fw	TATGTTTTAATAAGAGACATANNKGTAAATAGAATTACA
Cdif_KYxIw_I45-NNK-fw	AAGTATGTTTTAATAAGAGACNNKTTTGTAAATAGAATT
Cdif_KYxIw_D44-NNK-fw	ATTAAGTATGTTTTAATAAGANNKATATTTGTAAATAGA
Cdif_KYxIw_R43-NNK-fw	AAAATTAAGTATGTTTTAATANNKGACATATTTGTAAAT
Cdif_KYxIw_I42-NNK-fw	CATAAAATTAAGTATGTTTTANNKAGAGACATATTTGTA
Cdif_KYxIw_L41-NNK-fw	AATCATAAAATTAAGTATGTTNNKATAAGAGACATATTT
Cdif_KYxIw_V40-NNK-fw	TTAAATCATAAAATTAAGTATNNKTTAATAAGAGACATA
Cdif_KYxIw_Y39-NNK-fw	CTCTTAAATCATAAAATTAAGNNKGTTTTAATAAGAGAC
Cdif_KYxIw_K38-NNK-fw	GACCTCTTAAATCATAAAATTNNKTATGTTTTAATAAGA
Cdif_KYxIw_L75-rv2nd	TAAACTACAATACCGCCAAATATCATA
Cdif_KYxIw_C73-rv2nd	ACAATACCGCCAAATATCATATTTCTG
Cdif_KYxIw_R71-rv2nd	CCGCCAAATATCATATTTCTGAAAAAC
Cdif_KYxIw_I69-rv	AATATCATATTTCTGAAAAACTATATA
Cdif_KYxIw_Y67-rv	TTTCTGAAAAACTATATACTGTTTAGG
Cdif_KYxIw_Q65-rv	CTGAAAAACTATATACTGTTTAGGCAG
Cdif_KYxIw_V63-rv	TATATACTGTTTAGGCAGTCGTTCTTC
Cdif_KYxIw_Y61-rv	CTGTTTAGGCAGTCGTTCTTCAGAATA
Cdif_KYxIw_K59-rv	AGGCAGTCGTTCTTCAGAATATGTAAT
Cdif_KYxIw_L57-rv	TCGTTCTTCAGAATATGTAATTCTATT
Cdif_KYxIw_E55-rv	TTCAGAATATGTAATTCTATTTACAAA
Cdif_KYxIw_S53-rv	ATATGTAATTCTATTTACAAATATGTC
Cdif_KYxIw_T51-rv	AATTCTATTTACAAATATGTCTCTTAT
Cdif_KYxIw_R49-rv	ATTTACAAATATGTCTCTTATTAAAAC
Cdif_KYxIw_V47-rv	AAATATGTCTCTTATTAAAACATACTT
Cdif_KYxIw_I45-rv	GTCTCTTATTAAAACATACTTAATTTT
Cdif_KYxIw_R43-rv	TCTTATTAAAACATACTTAATTTTATG
Cdif_KYxIw_L41-rv	AACATACTTAATTTTATGATTTAAGAG
Cdif_KYxIw_Y39-rv	CTTAATTTTATGATTTAAGAGGTCTTT
Cdif_KYxIw_I37-rv	AATTTTATGATTTAAGAGGTCTTTTTT

Supplementary Table 5: Primers used for preparation of NGS library

Primer name	Sequence(5'-3')
P5-UDI0001-Rd1-TAG-gfp215-fw	AATGATACGGCGACCACCGAGATCTACACAGCGCTAGACACTCTTTCCCTACACGACGCTCTTCCGATCTNNNNNNTAGAGAGACCACATGGTCCTTCTT
P7-UDI0001-Rd2-ACC-FLAG2-8-rv	CAAGCAGAAGACGGCATACGAGATAACCGCGGGTGACTGGAGTTCAGACGTGTGCTCTTCCGATCTNNNNNNACCTTTGTCGTCGTCGTCTTTATA
P7-UDI0001-Rd2-AGC-FLAG2-8-rv	CAAGCAGAAGACGGCATACGAGATAACCGCGGGTGACTGGAGTTCAGACGTGTGCTCTTCCGATCTNNNNNNAGCTTTGTCGTCGTCGTCTTTATA
P7-UDI0001-Rd2-AGG-FLAG2-8-rv	CAAGCAGAAGACGGCATACGAGATAACCGCGGGTGACTGGAGTTCAGACGTGTGCTCTTCCGATCTNNNNNNAGGTTTGTCGTCGTCGTCTTTATA
P7-UDI0001-Rd2-CTC-FLAG2-8-rv	CAAGCAGAAGACGGCATACGAGATAACCGCGGGTGACTGGAGTTCAGACGTGTGCTCTTCCGATCTNNNNNNCTCTTTGTCGTCGTCGTCTTTATA
P7-UDI0001-Rd2-CTG-FLAG2-8-rv	CAAGCAGAAGACGGCATACGAGATAACCGCGGGTGACTGGAGTTCAGACGTGTGCTCTTCCGATCTNNNNNNCTGTTTGTCGTCGTCGTCTTTATA
P7-UDI0001-Rd2-TGC-FLAG2-8-rv	CAAGCAGAAGACGGCATACGAGATAACCGCGGGTGACTGGAGTTCAGACGTGTGCTCTTCCGATCTNNNNNNTGCTTTGTCGTCGTCGTCTTTATA
P7-UDI0001-Rd2-TTA-FLAG2-8-rv	CAAGCAGAAGACGGCATACGAGATAACCGCGGGTGACTGGAGTTCAGACGTGTGCTCTTCCGATCTNNNNNNTTATTTGTCGTCGTCGTCTTTATA
P7-UDI0001-Rd2-TTG-FLAG2-8-rv	CAAGCAGAAGACGGCATACGAGATAACCGCGGGTGACTGGAGTTCAGACGTGTGCTCTTCCGATCTNNNNNNTTGTTTGTCGTCGTCGTCTTTATA

Supplementary Table 6: MD simulation checklist.

**Reliability and reproducibility checklist for molecular dynamics simulations**<br>***All boxes must be marked YES by acceptance unless an N/A option is available**	**Yes**	**N/A**	**Response**<br>**(Please state where this information can be found in the text)**
**1. Convergence of simulations and analysis**			
1a. Is an evaluation presented in the text to show that the property being measured has equilibrated in the simulations (*e.g.* time-course analysis)?	☒		See ED Fig. 7b
1b. Then, is it described in the text how simulations are split into equilibration and production runs and how much data were analyzed from production runs?	☒		See Methods: MD simulation Set-up
1c. Are there at least 3 simulations per simulation condition with statistical analysis?	☒		10 replica simulations per condition each. See Methods: MD simulation setup.
1d. Is evidence provided in the text that the simulation results presented are independent of initial configuration?	☒		Evidence is provided in Figure 8a, showing that a wide conformational space around the cryo-EM model is explored by CliM NC.
**2. Connection to experiments**			
2a. Are calculations provided that can connect to experiments (*e.g.* loss or gain in function from mutagenesis, binding assays, NMR chemical shifts, J-couplings, SAXS curves, interaction distances or FRET distances, structure factors, diffusion coefficients, bulk modulus and other mechanical properties, *etc*.)?	☒		Mutagenesis experiments of stalling critical residue L75 (Fig. 7) were complemented and compared with MD simulations (Fig. 8a).
**3. Method choice**			
3a. Is it described in the text what force field and water model are used and why?	☒		See Methods: MD simulation Set-up
3b. Do simulations contain membranes, membrane proteins, intrinsically disordered proteins, glycans, nucleic acids, polymers, or cryptic ligand binding?	☒	☐	Nucleic acids

If 3b is **YES**, are enhanced sampling methods used?	☐	☒	Response not needed if **N/A**
If enhanced sampling methods are used, are the convergence criteria clearly stated?	☐		
If 3b is **YES**, is it explained in the text why or why not enhanced sampling methods are used?	☐		
**4. Code and reproducibility**			
4a. Is a table provided describing the system setup, such as simulation box dimensions, total number of atoms, total number of water molecules, salt concentration, lipid composition (number of molecules and type)?	☒		See Methods: MD simulation setup.
4b. Is it described in the text what simulation and analysis software and which versions are used?	☒		See Methods: MD simulation setup.
4c. Are initial coordinate and simulation input files and a coordinate file of the final output provided as supplementary files or in a public repository?	☒		https://doi.org/10.5281/zenodo.17779011
4d. Is there custom code or custom force field parameters?	☐	☒	Response not needed if **N/A**
If **YES**, are they provided as supplementary profiles or in a public repository?	☐		

Supplementary References

1. Syroegin, E. A., Aleksandrova, E. V & Polikanov, Y. S. Insights into the ribosome function from the structures of non-arrested ribosome-nascent chain complexes. *Nat. Chem.* 15, 143–153 (2023).

2. Gersteuer, F. *et al.* The SecM arrest peptide traps a pre-peptide bond formation state of the ribosome. *Nat. Commun.* 15, 2431 (2024).

3. Su, T. *et al.* The force-sensing peptide VemP employs extreme compaction and secondary structure formation to induce ribosomal stalling. *Elife* 6, (2017).

4. van der Stel, A.-X. *et al.* Structural basis for the tryptophan sensitivity of TnaC-mediated ribosome stalling. *Nat. Commun.* 12, 5340 (2021).

5. Matheisl, S., Berninghausen, O., Becker, T. & Beckmann, R. Structure of a human translation termination complex. *Nucleic Acids Res.* 43, 8615–26 (2015).

6. Aleksandrova, E. V *et al.* Mechanism of release factor-mediated peptidyl-tRNA hydrolysis on the ribosome. *Science* 388, eads9030 (2025).

7. Beckert, B. *et al.* Structural and mechanistic basis for translation inhibition by macrolide and ketolide antibiotics. *Nat. Commun.* 12, 4466 (2021).
