# Supplementary material for: Diverse mechanisms of translation arrest by a Clostridia ribosome stalling peptide CliM
Source: Nat Commun. 2026 May 18;17:4202. doi: 10.1038/s41467-026-72673-5 (PMC13183943; doi:10.1038/s41467-026-72673-5)
Supplement: Supplementary file 2 — Description of Additional Supplementary Files [file 41467_2026_72673_MOESM2_ESM.pdf]

Description of Additional Supplementary Files

File name: Supplementary Movie 1
Description: Cryo-EM map of CliM-SRC stalling at the stop codon of CliM, including a section that reveals the quality of the density (transparent surface) for the nascent chain (blue), P-tRNA (purple) and the release factor (RF, green) together with the molecular model. The final part of the video shows an overlay of an active RF (PDB ID 9MTP[28]) against the RF from the CliM-SRC, which illustrates an overlap of Q230 of the active RF with the penultimate residue from CliM. This clash results in a displaced GGQ loop of the RF in CliM.
