# Supplementary material for: Diverse mechanisms of translation arrest by a Clostridia ribosome stalling peptide CliM
Source: Nat Commun. 2026 May 18;17:4202. doi: 10.1038/s41467-026-72673-5 (PMC13183943; doi:10.1038/s41467-026-72673-5)
Supplement: Supplementary file 4 — Reporting Summary [file 41467_2026_72673_MOESM4_ESM.pdf]

nature portfolio

Corresponding author(s): Daniel Wilson and Shinobu Chiba

Last updated by author(s): Apr 7, 2026

Reporting Summary

Nature Portfolio wishes to improve the reproducibility of the work that we publish. This form provides structure for consistency and transparency in reporting. For further information on Nature Portfolio policies, see our Editorial Policies and the Editorial Policy Checklist.

Statistics

For all statistical analyses, confirm that the following items are present in the figure legend, table legend, main text, or Methods section.

n/a	Confirmed	
☐	☒	The exact sample size (*n*) for each experimental group/condition, given as a discrete number and unit of measurement
☐	☒	A statement on whether measurements were taken from distinct samples or whether the same sample was measured repeatedly
☒	☐	The statistical test(s) used AND whether they are one- or two-sided<br>*Only common tests should be described solely by name; describe more complex techniques in the Methods section.*
☒	☐	A description of all covariates tested
☒	☐	A description of any assumptions or corrections, such as tests of normality and adjustment for multiple comparisons
☐	☒	A full description of the statistical parameters including central tendency (e.g. means) or other basic estimates (e.g. regression coefficient) AND variation (e.g. standard deviation) or associated estimates of uncertainty (e.g. confidence intervals)
☒	☐	For null hypothesis testing, the test statistic (e.g. *F*, *t*, *r*) with confidence intervals, effect sizes, degrees of freedom and *P* value noted<br>*Give P values as exact values whenever suitable.*
☒	☐	For Bayesian analysis, information on the choice of priors and Markov chain Monte Carlo settings
☒	☐	For hierarchical and complex designs, identification of the appropriate level for tests and full reporting of outcomes
☒	☐	Estimates of effect sizes (e.g. Cohen's *d*, Pearson's *r*), indicating how they were calculated

Our web collection on statistics for biologists contains articles on many of the points above.

Software and code

Policy information about availability of computer code

Data collection	CryoEM data were collected using the EPU softare version 3.2.0.4775REL (FEI, Netherlands) using AutoCTF function of Sherpa (version 2.11.1)
Data analysis	RELION v 4 with MotionCor2 v1.2.1 and CTFFIND 4.1.14 were used for processing micrographs, picking particles, classification and refining cryo-EM maps. RELION was used to calculate local resolution. Coot v0.9.8.92 for model building and ServalCat v0.3.1 with REFMAC 5 v5.8.0415 for model refinement and statistics, with structural restraints generated by aceDRG. Figures were generated using ChimeraX v1.9. Validation was performed using Phenix 1.20–4487.<br>MD simulations were prepared, performed and analyzed using GROMACS 2025 with the implemented LINCS and GENION versions. For preparation WHATIF 20071220-093 was used. Overlap volume calculation for MD trajectories was scripted in Python 3.13. Functional mode analysis was conducted with a GROMACS branch that can be downloaded at https://www3.mpibpc.mpg.de/groups/de_groot/fma.html. Figures with MD simulation results were created with R 4.5.1 and ChimeraX 1.10.1.

For manuscripts utilizing custom algorithms or software that are central to the research but not yet described in published literature, software must be made available to editors and reviewers. We strongly encourage code deposition in a community repository (e.g. GitHub). See the Nature Portfolio guidelines for submitting code & software for further information.

Data

Policy information about availability of data

All manuscripts must include a data availability statement. This statement should provide the following information, where applicable:

- Accession codes, unique identifiers, or web links for publicly available datasets
- A description of any restrictions on data availability
- For clinical datasets or third party data, please ensure that the statement adheres to our policy

The raw sequencing reads from the deep mutational scanning have been deposited in the DDBJ under BioProject accession number PRJDB39765. The cryo-electron microscopy maps and the respective coordinates for electron-microscopy-based model have been deposited in the EMDataBank and PDB under the accession numbers EMD50855, EMD50856 and E MD50858 and PDB ID 9FY1, 9FY2 and 9FY3.
MD simulation input files, final coordinates and analyses are available on zenodo.org (https://doi.org/10.5281/zenodo.17779011).
The following structures from the PDB were used for resaons of figure preparation and comparison: 9MTP, 8QOA, 5NWY, 7O19, 5A81, 8CVK and 7NSO.

Research involving human participants, their data, or biological material

Policy information about studies with human participants or human data. See also policy information about sex, gender (identity/presentation), and sexual orientation and race, ethnicity and racism.

Reporting on sex and gender	N/A
Reporting on race, ethnicity, or other socially relevant groupings	N/A
Population characteristics	N/A
Recruitment	N/A
Ethics oversight	N/A

Note that full information on the approval of the study protocol must also be provided in the manuscript.

Field-specific reporting

Please select the one below that is the best fit for your research. If you are not sure, read the appropriate sections before making your selection.

☒ Life sciences ☐ Behavioural & social sciences ☐ Ecological, evolutionary & environmental sciences

For a reference copy of the document with all sections, see nature.com/documents/nr-reporting-summary-flat.pdf

Life sciences study design

All studies must disclose on these points even when the disclosure is negative.

Sample size	No sample size calculation was performed. The sample size was selected on the basis of a two-day data collection, which was chosen to obtain sufficient number of particles to bring the resolution of the resulting complexes towards 2A resolution.
Data exclusions	Micrographs with low estimated resolution or poorly fitted CTFs were discarded, as were particles that clustered into poorly defined classes during 2D and 3D classification.
Replication	For b-galactosidase assay, three or six biological replicates were averaged.<br>For in vitro translation arrest assay and toeprinting, one of at least two biological replicates are presented.
Randomization	For 3D refinement in RELION, particles are randomly placed in one of two subsets. These subsets are maintained for CTF refinement. Otherwise, no randomization was performed. For the molecular dynamics simulations, to obtain statistical uncertainties, 1000 subsets of conformations were randomly selected and the analysis was repeated on each subset.
Blinding	No blinding was performed as blinding is not possible or not applicable for the experiments because the identity of the analyzed sample was known.

Reporting for specific materials, systems and methods

We require information from authors about some types of materials, experimental systems and methods used in many studies. Here, indicate whether each material, system or method listed is relevant to your study. If you are not sure if a list item applies to your research, read the appropriate section before selecting a response.

Materials & experimental systems

n/a	Involved in the study
☐	☒ Antibodies
☒	☐ Eukaryotic cell lines
☒	☐ Palaeontology and archaeology
☒	☐ Animals and other organisms
☒	☐ Clinical data
☒	☐ Dual use research of concern
☒	☐ Plants

Methods

n/a	Involved in the study
☒	☐ ChIP-seq
☒	☐ Flow cytometry
☒	☐ MRI-based neuroimaging

Antibodies

Antibodies used	anti-GFP antibody (Wako, 012-22541, clone mFX75)
Validation	The specificity of the anti-GFP antibody was validated by Western blotting in this study, showing that the size of the proteins detected by the antibodies were equivalent to the size of target proteins synthesized in vitro using specific DNA templates encoding the target proteins.

Plants

Seed stocks	N/A
Novel plant genotypes	N/A
Authentication	N/A
